# Breakroom Culture and Nurse Burnout: A Multilevel Moderated Mediation Analysis

**DOI:** 10.1155/jonm/5545718

**Published:** 2026-08-21

**Authors:** Yanjun Wu, Yanyan Lu, Wubin He

**Affiliations:** ^1^ Department of Thoracic Surgery, The First Affiliated Hospital of Jinzhou Medical University, Jinzhou, China, jzmu.edu.cn; ^2^ School of Nursing, Jinzhou Medical University, Jinzhou, China, jzmu.edu.cn; ^3^ Key Laboratory of Surgery of Liaoning Province of the First Affiliated Hospital of Jinzhou Medical University, Jinzhou, China

**Keywords:** breakroom culture, burnout, China, cross-sectional study, multilevel analysis, nurses, nursing management, perceived organizational support, team-level work engagement

## Abstract

**Aim:**

To examine whether perceived organisational support (POS) mediates the association between breakroom culture and nurse burnout (low psychological well‐being and disengagement) and whether unit‐level teamwork engagement moderates this mediation.

**Background:**

Nurse burnout threatens care quality and workforce retention globally. Informal social resources embedded in the everyday work environment, such as breakroom culture (shared supportive peer interactions during rest periods), remain underexplored—particularly in nonWestern healthcare contexts. Grounded in the Job Demands–Resources theory and Organisational Support Theory, this study examined the mediating role of POS and the moderating role of unit‐level team work engagement in the breakroom culture–burnout relationship.

**Methods:**

A cross‐sectional multilevel study was conducted with 720 nurses nested in 36 units across three Chinese tertiary hospitals. Breakroom culture was measured using a contextually validated five‐item scale; POS with the revised Survey of POS; low psychological well‐being (reverse‐scored WHO‐5, as an indicator of exhaustion‐related distress) and disengagement (adapted cynicism subscale) as individual‐level outcomes. Unit‐level team work engagement was measured using the UWES‐9‐T. Multilevel structural equation modelling with latent decomposition and 5000 bootstrap resamples tested the hypothesised multilevel moderated mediation model (unit‐level moderator, individual‐level mediator and outcomes).

**Results:**

Breakroom culture was positively associated with POS (*β* = 0.39, *p* < 0.001), which in turn was negatively associated with low psychological well‐being (*β* = −0.46, *p* < 0.001) and disengagement (*β* = −0.41, *p* < 0.001). Indirect effects via POS were significant for low psychological well‐being (ab = −0.18, 95% CI [–0.24, −0.13]) and disengagement (ab = −0.16, 95% CI [–0.21, −0.12]), with no residual direct effects. Team‐level work engagement moderated the first stage of mediation (βinteraction = 0.15, *p* = 0.003); the indirect effects were significantly stronger in units with higher team work engagement (indices of moderated mediation: −0.06 and −0.05).

**Conclusion:**

A supportive breakroom culture is associated with reduced nurse burnout through enhanced POS—an informal‐organisational support pathway amplified by positive team‐level work engagement. Due to the cross‐sectional design, causal claims cannot be established from this study.

**Implications for Nursing Management:**

Nurse managers should reconceptualise breakrooms as strategic recovery spaces, apply low‐cost environmental upgrades, and model relational leadership during breaks. Written task guidelines should protect break time from nonclinical chores. Routine monitoring of team‐level work engagement using validated tools (e.g., UWES‐9‐T) is recommended. Healthcare institutions should embed relational well‐being metrics into quality dashboards and accreditation standards.

## 1. Introduction

Nurse burnout—a psychological syndrome characterised by low psychological well‐being (exhaustion‐related distress) and disengagement (cynicism) [1]—remains a persistent global challenge associated with compromised healthcare quality, patient safety and workforce sustainability [2, 3]. High prevalence rates are consistently reported across diverse settings, including Asia, underscoring the need for effective mitigation strategies [4]. Although healthcare organisations often implement formal, top‐down interventions, the impact of such structural approaches can be constrained by systemic resources and organisational inertia [5]. Consequently, scholarly and managerial attention has increasingly turned to the protective potential of informal social resources embedded within the everyday work environment [6].

Within the high‐demand nursing ecosystem, the staff breakroom constitutes a critical liminal space—a transitional area distinct from direct clinical duties where informal peer interaction, unstructured emotional exchange and momentary psychological recovery occur. The quality of mutual support and social exchange in this specific context, termed breakroom culture, may represent a potent yet underexamined job resource. Breakroom culture is defined as the shared perception of supportive, caring and collegial peer interactions occurring specifically within the staff breakroom during rest periods—a context‐specific manifestation of informal coworker support [7] grounded in social exchange and organisational support theory (OST) [8]. However, empirical evidence directly linking this microlevel, informal social phenomenon to nurse burnout remains scarce, particularly in non‐Western healthcare contexts [9]. Moreover, the psychological mechanisms through which a positive breakroom culture may operate are poorly understood, limiting the development of targeted, contextually appropriate management strategies.

In Chinese tertiary hospitals, preliminary qualitative feedback from our pilot interviews revealed that nurses commonly perceive breakrooms as “another workspace” rather than a sanctuary—a finding that underscores the need to reconceptualise these liminal spaces as strategic resources for well‐being.

Although burnout and psychological well‐being are distinct constructs, they represent complementary facets of occupational health. Burnout captures the negative endpoint of chronic resource depletion, characterised by disengagement and diminished professional efficacy [1], while psychological well‐being, as measured by the World Health Organization‐5 (WHO‐5), encompasses positive dimensions of mental health including positive mood, vitality and general interest [10]. The WHO‐5 Well‐Being Index, as a measure of positive mental health, offers a uniquely sensitive indicator of the erosion of psychological well‐being that often accompanies and may precede clinically significant burnout [10]. In healthcare populations, WHO‐5 scores have been prospectively associated with both concurrent burnout and subsequent sickness absence [11], supporting its utility as a standalone well‐being outcome in occupational health research. By examining breakroom culture’s association with both low psychological well‐being (WHO‐5) and disengagement (a core burnout dimension), this study captures the full spectrum of nurse psychological health—from diminished well‐being to established burnout attitudes—thereby providing a more comprehensive test of the informal‐organisational support pathway than either indicator alone would permit.

To address these gaps, this study integrates the Job Demands–Resources (JD‐R) theory [12] and OST [8] to propose a coherent model. We hypothesise that a supportive breakroom culture, as a social job resource, is positively associated with a nurse’s perceived organisational support (POS)—the global belief that the organisation values their contributions and cares about their well‐being [8, 13]. Breakroom culture is theoretically grounded in perceived coworker support theory [7], social exchange theory as embedded in OST [8], and the recovery and detachment literature [14]; it represents a context‐specific application of established interpersonal support constructs to the physical and social setting of the staff breakroom. According to OST, POS is shaped not only by formal policies but also by symbolic cues from the social environment [15]. Thus, supportive peer interactions in the breakroom may be interpreted by nurses as evidence of a caring organisational ethos, thereby contributing to elevated POS. In turn, POS is a well‐established buffer against the core dimensions of burnout [16].

We further propose that this indirect pathway is context‐dependent. Unit‐level team work engagement—the shared, collective experience of vigour, dedication, and absorption within a work team [17]—is hypothesised to moderate the first stage of mediation. In units with a strong, positive team work engagement, supportive acts in the breakroom align with and reinforce the prevailing normative environment, strengthening nurses’ attribution of such support to a broadly supportive organisation. Conversely, in units with poor team work engagement, positive breakroom interactions may be perceived as isolated exceptions, weakening their impact on organisational judgements [18]. The informal‐organisational support pathway is therefore expected to be most potent under positive team work engagement.

This study is situated within China’s hierarchical healthcare context, where power distance may influence access to formal support and informal relational networks (guanxi) carry exceptional significance for well‐being [19, 20]. Examining these cross‐level dynamics can provide actionable, contextually nuanced evidence for nurse leaders on how to synergistically cultivate cohesive formal teams and supportive informal spaces to mitigate burnout. Specifically, this study aims to examine (1) whether POS mediates the relationship between breakroom culture and nurse burnout (low psychological well‐being and disengagement) and (2) whether unit‐level team work engagement moderates the first stage of this mediation, thereby testing a multilevel moderated mediation model. The following hypotheses were formally tested:

H1: POS mediates the association between breakroom culture and low psychological well‐being (exhaustion‐related distress).

H2: POS mediates the association between breakroom culture and disengagement (cynicism).

H3a: Unit‐level team work engagement moderates the first stage of the indirect pathway (breakroom culture ⟶ POS), such that the indirect association with low well‐being is stronger in units with higher team work engagement.

H3b: The moderating effect described in H3a extends to disengagement as the outcome.

## 2. Methods

### 2.1. Study Design and Participants

**FIGURE 1:**
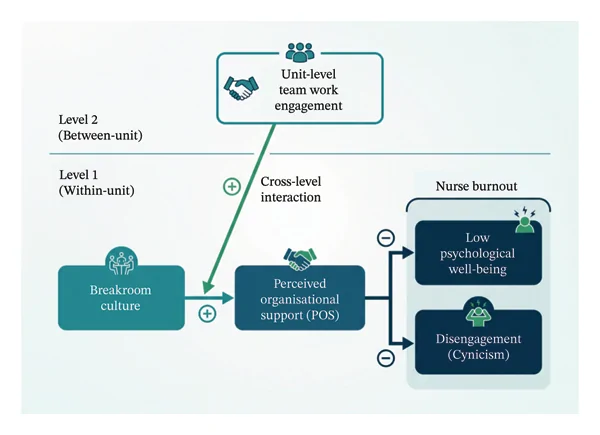
Hypothesised conceptual model.

A cross‐sectional multilevel design was employed, with individual nurses (Level 1) nested within clinical units (Level 2). The hypothesised conceptual model guiding this study is presented in Figure 1. The model specifies a cross‐level moderated mediation framework in which breakroom culture is indirectly associated with nurse burnout (low psychological well‐being and disengagement) through POS at the individual level, with unit‐level team work engagement moderating the first stage of this indirect pathway. Owing to the cross‐sectional nature of the data, claims regarding the direction of effects cannot be conclusively established; rather, the study tests theoretically derived associations using robust multilevel modelling techniques. Data were collected between March and October 2025 from three tertiary public hospitals in Liaoning Province, China. A convenience sample of 36 diverse clinical units (e.g., internal medicine, surgery, ICU and emergency) was recruited. All eligible full‐time registered nurses within those units were invited to participate. The final sample comprised 720 nurses from 36 units, with a mean cluster size of 20 nurses (range: 15–28), which is adequate for multilevel modelling. The study received ethical approval from the relevant institutional review boards. Written informed consent was obtained from all participants, with guarantees of anonymity and voluntary participation.

### 2.2. Ethical Approval

This study was conducted in accordance with the principles outlined in the Declaration of Helsinki. Ethical approval for the study protocol was obtained from the appropriate institutional research ethics committee prior to data collection. All participants provided written informed consent prior to participation, with guarantees of anonymity and voluntary withdrawal.

### 2.3. Measures and Cross‐Cultural Adaptation

All study instruments underwent a rigorous cross‐cultural adaptation process into Mandarin Chinese, following established guidelines involving forward/backward translation, expert panel review, and cognitive debriefing with frontline nurses [21]. This process ensured conceptual, semantic and operational equivalence. To mitigate potential common method bias, procedural remedies were implemented during data collection, including ensuring respondent anonymity, using sealed return boxes and randomising the order of scale items [22]. In adherence to open science principles, we utilized publicly available or adapted scales with permission terms for noncommercial scientific research, as detailed in Supporting Material 1.

Breakroom culture was assessed using a five‐item scale developed contextually for this study. Breakroom culture is defined as the shared perception of supportive, caring and collegial peer interactions occurring specifically within the staff breakroom during rest periods—a context‐specific manifestation of informal coworker support [7] grounded in social exchange and OST [8]. The scale was developed through a systematic six‐step validation process. Step 1: Based on the theoretical construct of perceived coworker support [7], we generated an initial pool of 12 items reflecting supportive interactions specifically within the breakroom setting. Step 2: Content validity was evaluated by a panel of five experts: two nursing managers (both with > 15 years of clinical and administrative experience), two senior clinical nurses (each with > 10 years of bedside practice), and one occupational health psychologist. Items with an Item‐level Content Validity Index (I‐CVI) below 0.78 were removed, resulting in eight retained items (S‐CVI/Ave = 0.94). Step 3: An exploratory factor analysis (EFA) on an independent pilot sample of 180 nurses (not included in the main study) revealed a unidimensional structure with factor loadings > 0.70, explaining 68.4% of the variance. Step 4: Three items with the lowest loadings or high cross‐loadings were deleted, yielding the final five‐item scale. Confirmatory factor analysis (CFA) in the main sample supported the unidimensional structure (CFI = 0.99, RMSEA = 0.03). Step 5: Discriminant validity was established against a separate 4‐item measure of general perceived coworker support [7]; a two‐factor model (breakroom culture and general coworker support) fitted significantly better than a one‐factor model (Δ*χ* ^2^ [1] = 47.2, *p* < 0.001), and the square root of the average variance extracted (AVE) for breakroom culture (0.80) exceeded its correlation with general coworker support (*r* = 0.51). Step 6: The scale demonstrated excellent internal consistency in this study (Cronbach’s *α* = 0.89; CR = 0.90; AVE = 0.64). Full psychometric details are provided in Supporting Material 2, Table S1. Aggregation statistics for breakroom culture at the unit level showed acceptable reliability (ICC [2] = 0.78), supporting its use as a unit‐level construct in between‐level analyses. As a study‐specific, contextually validated measure, independent validation in future research is encouraged.

Perceived organizational support (POS) was measured using the revised public‐domain version of the Survey of POS (SPOS), which is freely available for use without permission [23]. A sample item is “*The organization strongly considers my goals and values.*” Responses were given on a five‐point Likert scale (1 = Strongly Disagree to 5 = Strongly Agree). The scale showed high reliability (*α* = 0.92).

Burnout was evaluated using two dimensions. Low psychological well‐being, as an indicator of exhaustion‐related distress, was assessed using the WHO‐5 Well‐Being Index [10], a widely used and freely available measure of positive mental well‐being. The WHO‐5 was designed to measure subjective psychological well‐being, not occupational exhaustion per se. Consistent with its established use as both a positive well‐being measure and, when reverse‐scored, a validated indicator of psychological distress in occupational and healthcare populations [10, 24], the five‐item scale was reverse‐scored so that lower scores indicate lower well‐being/higher exhaustion‐related distress. Convergent validity with the emotional exhaustion subscale of the Maslach Burnout Inventory‐General Survey (MBI‐GS; [25]) was *r* = −0.73 (*p* < 0.001) in a separate validation subsample (*N* = 120). The MBI‐GS was selected for this correlational validation as the most widely recognised international standard for burnout assessment. The MBI‐GS is a copyrighted instrument (Mind Garden, Inc.); its items are not reproduced in this manuscript or its supporting materials. Additionally, supporting mediation analysis on this same subsample confirmed that the indirect effect pattern (breakroom culture ⟶ POS ⟶ outcome) was comparable when the outcome was operationalised using reverse‐scored WHO‐5 versus the MBI‐GS emotional exhaustion subscale (Δab = −0.02, 95% CI [–0.07, 0.04]; see Supporting Material 3 for full details), supporting the sensitivity of the WHO‐5 to exhaustion‐related distress while acknowledging it measures the broader construct of psychological well‐being. The scale showed good reliability in this study (*α* = 0.85). Disengagement (conceptualised as cynicism) was measured using a four‐item cynicism subscale adapted from publicly available burnout research literature [26], which captures negative and detached attitudes towards one’s work (e.g., “*I have become more cynical about whether my work contributes anything*”). Responses were recorded on a five‐point Likert scale (1 = Strongly Disagree to 5 = Strongly Agree). The adapted measure showed good reliability (*α* = 0.82). Multilevel MCFA (MCFA) (Supporting Material 2, Table S2 and Part A) supported the discriminant validity of low psychological well‐being and disengagement.

Team‐level work engagement was measured as a unit‐level (Level 2) construct using the nine‐item team version of the Utrecht Work Engagement Scale (UWES‐9‐T) [17], reflecting the shared, collective experience of vigour, dedication, and absorption within the work team. We operationalised team‐level work engagement through a reference‐shift composition model [27] in which nurses rated their immediate work team using the stem “*As a team, we…*” on items like “*…are enthusiastic about our jobs*” on a seven‐point frequency scale (0 = Never to 6 = Always).

We selected this focused measurement strategy over broader team climate instruments such as the Team Climate Inventory (TCI; [28]) for three theoretical and practical reasons. First, our moderation hypothesis is specifically concerned with the affective‐motivational coherence between informal peer support (breakroom) and the formal team environment—that is, whether the shared emotional quality of team interactions amplifies nurses’ attribution of breakroom support to organisational care. The UWES‐9‐T’s vigour‐dedication‐absorption dimensions directly capture this affective‐motivational core, whereas the TCI’s dimensions of participative safety, support for innovation, task orientation and vision [28], while important for team functioning, are less theoretically proximal to the attribution mechanism under investigation. Second, the UWES‐9‐T has been extensively validated in Chinese healthcare populations, demonstrating robust psychometric properties and cultural appropriateness [17]. Third, its brevity (9 items) reduces respondent burden—a practical consideration when conducting research with frontline clinical staff during work hours. We acknowledge that this is a focused measurement strategy involving a trade‐off: we gain theoretical precision for the specific affective‐motivational moderation mechanism under investigation at the cost of not capturing other dimensions of team climate (e.g., participative safety and task orientation).

Aggregation of individual ratings to the unit level was statistically justified. The mean within‐group agreement index (rwg(j)) was 0.88 (range: 0.72–0.96 across units); only one unit fell below the conventional 0.70 threshold for breakroom culture, and sensitivity analysis excluding this unit yielded an identical pattern of results (Supporting Material 2, Table S2, Part B). The intraclass correlation coefficient (ICC [1]) was 0.40 (*p* < 0.001), confirming significant between‐unit variance. The reliability of the unit means (ICC [2]) was 0.82. The aggregated scale reliability was excellent (*α* = 0.93).

This focused measurement strategy necessarily excludes other dimensions of team climate as conceptualised in broader frameworks [28]. We therefore explicitly do not claim to have measured team climate in its full multidimensional sense, and we have labelled our construct as “team‐level work engagement” throughout to accurately reflect the instrument’s scope. Future studies using comprehensive climate instruments such as the TCI can determine whether the observed moderating effect generalises across all climate dimensions or is specific to the affective‐motivational core captured by team work engagement.

Covariates included at the individual level were age, gender, education level, total years of nursing experience, tenure in the current unit and shift pattern (day‐only vs. rotating shifts). Given that 74% of the sample worked rotating shifts (including nights), we conducted supporting analyses to examine whether shift pattern moderated any of the hypothesised paths. Shift pattern was entered as a moderator of the breakroom culture ⟶ POS and POS ⟶ outcome paths; no significant interaction effects were detected, and the pattern of conditional indirect effects remained stable. These results are available in Supporting Material 3. Covariates were selected based on prior research indicating their potential associations with burnout and organisational perceptions [e.g., 4, 12].

The complete item lists for all measurement scales used in this study, presented in both English and the adapted Mandarin Chinese versions, are provided in Supporting Material 1.

### 2.4. Statistical Analysis

Data analysis was conducted using Mplus 8.6 with robust maximum likelihood (MLR) estimation. Missing data were minimal (overall < 2% across all items) and were handled using full information maximum likelihood (FIML) estimation under the missing‐at‐random (MAR) assumption, implemented through the MLR estimator in Mplus 8.6.

Preliminary Analysis: Descriptive statistics, scale reliabilities and bivariate correlations were computed. ICCs were calculated to quantify between‐unit variance for all individual‐level variables, justifying the multilevel approach.

Measurement Validation: A multilevel MCFA tested the discriminant validity of the four‐factor measurement model (breakroom culture, POS, low psychological well‐being and disengagement) at both within‐ and between‐levels.

Hypothesis Testing: The hypothesised cross‐level moderated mediation model was tested using multilevel structural equation modelling (MSEM). The full Mplus syntax is provided in Supporting Material 2. Following established procedures for 2–1‐1 mediation with a Level‐2 moderator [29], latent variable decomposition was applied to separate the within‐ and between‐level components of the mediator (POS). The model tested:

Level 1 (Within‐unit): The mediation path: Breakroom Culture ⟶ POS ⟶ Burnout (separately for Low Well‐Being and Disengagement).

Level 2 (Between‐unit): Unit‐level Team Work Engagement was entered as a moderator of the Breakroom Culture ⟶ POS path (a cross‐level interaction).

Direct, indirect, and conditional indirect effects were tested using bias‐corrected bootstrap confidence intervals with 5000 resamples. Model fit was evaluated using standard criteria: CFI/TLI ≥ 0.95, RMSEA ≤ 0.06 and SRMR (within < 0.08, between < 0.10) [30].

Robustness Checks: To assess the robustness of our findings, we compared the hypothesised model against several alternative specifications. All alternative models exhibited an inferior fit to the hypothesised model (see Supporting Material 2, Tables S4–S5 for complete path coefficients and fit comparisons). Additionally, we examined whether the pattern of conditional indirect effects was sensitive to the inclusion of covariates; all parameter estimates remained stable in direction, magnitude and statistical significance when covariates were omitted.

## 3. Results

### 3.1. Sample Characteristics and Preliminary Analyses

The sample comprised 720 nurses, predominantly female (92.5%), with a mean age of 32.4 years (SD = 7.1). Participants had an average of 8.6 years (SD = 6.3) of total nursing experience and 5.2 years (SD = 4.4) of tenure in their current unit. A detailed breakdown of demographic and work‐related characteristics is provided in Supporting Material 2, Table S3.

Descriptive statistics, reliability estimates and intercorrelations for all study variables are presented in Table 1. All scales demonstrated good to excellent internal consistency (*α* = 0.82–0.93). ICCs were significant for breakroom culture (0.38, *p* < 0.001), POS (0.41, *p* < 0.001), low psychological well‐being (0.33, *p* < 0.001) and disengagement (0.29, *p* < 0.001), confirming substantial between‐unit variance and justifying multilevel analysis. At the individual level, breakroom culture was positively correlated with POS (*r* = 0.42, *p* < 0.001) and negatively correlated with low well‐being (*r* = −0.36, *p* < 0.001) and disengagement (*r* = −0.33, *p* < 0.001). POS was strongly negatively correlated with both low well‐being (*r* = −0.49, *p* < 0.001) and disengagement (*r* = −0.45, *p* < 0.001).

**TABLE 1:** Descriptive statistics, reliability and intercorrelations among study variables.

Variables	M	SD	*α* (Within)	*α* (Between)	ICC (1)	1	2	3	4
Level‐1 (*N* = 720)									
1. Breakroom Culture	3.85	0.76	0.89	0.91	0.38^∗∗∗^	—			
2. POS	3.78	0.81	0.92	0.94	0.41^∗∗∗^	0.42^∗∗∗^	—		
3. Low Psychological Well‐Being (WHO‐5‐R)	2.98	0.79	0.85	—	0.33^∗∗∗^	−0.36^∗∗∗^	−0.49^∗∗∗^	—	
4. Disengagement (Cynicism)	2.91	0.83	0.82	—	0.29^∗∗∗^	−0.33^∗∗∗^	−0.45^∗∗∗^	0.61^∗∗∗^	—
Level‐2 (*J* = 36)									
5. Team‐Level Work Engagement (UWES‐9‐T)	4.05	0.71	—	0.93	0.40^∗∗∗^	0.39^∗∗^	0.45^∗∗^	−0.35^∗∗^	−0.31^∗^

*Note:* M = mean; *α* = Cronbach’s alpha; ICC (1) = intraclass correlation coefficient. *α* (Between) represents the reliability of the aggregated unit‐level mean for team‐level work engagement; for individual‐level variables, between‐level reliability is not applicable. Level‐1 correlations are within‐level. Level‐2 correlations are between‐unit aggregates.

Abbreviation: SD = standard deviation.

^∗^ *p* < 0.05.

^∗∗^ *p* < 0.01.

^∗∗∗^ *p* < 0.001.

### 3.2. Measurement Model and Hypothesized Model Fit

The MCFA for the four‐factor measurement model (breakroom culture, POS, low psychological well‐being and disengagement) demonstrated excellent fit to the data: CFI = 0.97, TLI = 0.96, RMSEA = 0.04, SRMRwithin = 0.03, SRMRbetween = 0.05. All standardized factor loadings were significant at *p* < 0.001 and exceeded 0.70 at both within‐ and between‐levels, supporting the discriminant validity of the four constructs. Detailed loading estimates, composite reliabilities, and average variance extracted are provided in Supporting Material 2, Table S2 (Part A).

The aggregated scale reliability for unit‐level team work engagement was excellent (*α* = 0.93). Statistical justification for aggregating both breakroom culture and team work engagement to the unit level is documented in Supporting Material 2, Table S2 (Part B). All aggregation indices met recommended thresholds.

The hypothesised cross‐level moderated mediation model also exhibited strong fit to the data: *χ* ^2^(128) = 235.17, *p* < 0.001; CFI = 0.970; TLI = 0.962; RMSEA = 0.039 (90% CI [0.031–0.047]); SRMRwithin = 0.040; SRMRbetween = 0.055. This model fit significantly better than all alternative specifications tested (see Section 3.4 and Supporting Material 2, Table S5).

### 3.3. Tests of Hypotheses

The results of the MSEM analysis are summarised in Table 2. At Level 1, breakroom culture was positively associated with POS (*β* = 0.39, *p* < 0.001). POS, in turn, was negatively associated with both low psychological well‐being (*β* = −0.46, *p* < 0.001) and disengagement (*β* = −0.41, *p* < 0.001). The direct paths from breakroom culture to low well‐being (*β* = −0.05, *p* = 0.220) and disengagement (*β* = −0.04, *p* = 0.298) were nonsignificant.

**TABLE 2:** Results of multilevel moderated mediation analysis.

Path/Effect	Estimate (b)	SE	95% BCa CI	Supported?
Direct Paths (Level 1)				
Breakroom Culture ⟶ POS	0.39^∗∗∗^	0.05	[0.28, 0.50]	Yes
POS ⟶ Low Well‐Being	−0.46^∗∗∗^	0.06	[−0.58, −0.34]	Yes
POS ⟶ Disengagement	−0.41^∗∗∗^	0.05	[−0.52, −0.30]	Yes
Breakroom Culture ⟶ Low Well‐Being (direct)	−0.05	0.04	[−0.14, 0.04]	No
Breakroom Culture ⟶ Disengagement (direct)	−0.04	0.04	[−0.12, 0.05]	No
Indirect Effects (Unconditional)				
H1: Br. Culture ⟶ POS ⟶ Low Well‐Being	−0.18^∗^	0.03	[−0.24, −0.13]	Yes
H2: Br. Culture ⟶ POS ⟶ Disengagement	−0.16^∗^	0.02	[−0.21, −0.12]	Yes
Conditional Indirect Effects (by Team‐Level Work Engagement)				
For Low Well‐Being:				
Low Team Work Engagement (−1 SD)	−0.12^∗∗∗^	0.03	[−0.18, −0.07]	
Average Team Work Engagement	−0.18^∗∗∗^	0.03	[−0.24, −0.13]	
High Team Work Engagement (+1 SD)	−0.24^∗∗∗^	0.04	[−0.32, −0.17]	
For Disengagement:				
Low Team Work Engagement (−1 SD)	−0.11^∗∗∗^	0.02	[−0.16, −0.07]	
Average Team Work Engagement	−0.16^∗∗∗^	0.02	[−0.21, −0.12]	
High Team Work Engagement (+1 SD)	−0.21^∗∗∗^	0.03	[−0.28, −0.15]	
Index of Moderated Mediation				
For Low Well‐Being (H3a)	−0.06^∗∗^	0.02	[−0.11, −0.03]	Yes
For Disengagement (H3b)	−0.05^∗^	0.02	[−0.09, −0.02]	Yes

*Note:* BCa CI = bias‐corrected and accelerated bootstrap confidence interval (5000 resamples). All models controlled for covariates.

^∗^ *p* < 0.05.

^∗∗^ *p* < 0.01.

^∗∗∗^ *p* < 0.001.

Bootstrap analyses confirmed significant negative indirect associations of breakroom culture with low psychological well‐being (ab = −0.18, 95% BCa CI [–0.24, −0.13]) and disengagement (ab = −0.16, 95% BCa CI [–0.21, −0.12]) via POS. These findings indicate that the informal peer support occurring in breakroom spaces is not merely a microlevel social phenomenon but a conduit through which nurses infer broader organisational care—a mechanism that has received limited empirical attention in the nursing management literature.

In practical terms, these indirect associations indicate that a one‐unit increase in breakroom culture corresponds to a 0.18‐unit reduction in low well‐being scores through enhanced POS at the mean level of team work engagement. While this effect is modest in absolute magnitude, it is comparable to or larger than typical indirect effects reported in multilevel organisational health research (see Discussion 4.1). Importantly, the indirect association more than doubles from low to high team work engagement contexts (from −0.12 to −0.24), suggesting a meaningful contextual amplification.

**FIGURE 2:**
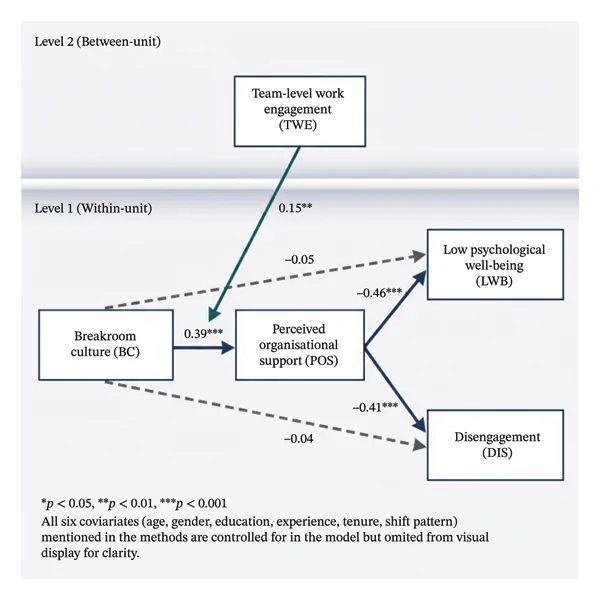
Multilevel moderated mediation model with standardised path coefficients.

**FIGURE 3:**
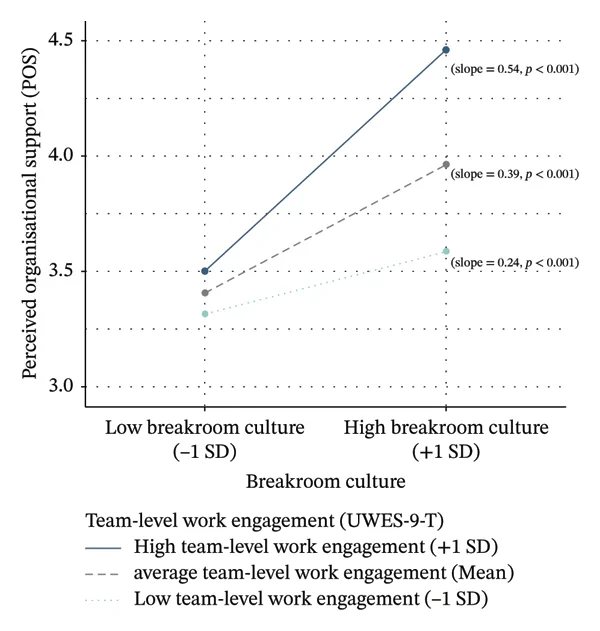
Moderating effect of unit‐level team work engagement on the relationship between breakroom culture and perceived organisational support (POS).

Supporting H3, a significant cross‐level interaction was found between breakroom culture (aggregated to the unit level) and unit‐level team work engagement on POS (βinteraction = 0.15, *p* = 0.003). Figure 2 presents a schematic of the overall moderated mediation model with standardised path coefficients. Simple slopes analysis (Figure 3) revealed that the positive association between breakroom culture and POS was strongest in units with high team work engagement (simple slope = 0.54, *p* < 0.001), moderate in units with average engagement (slope = 0.39, *p* < 0.001), and weakest—yet still significant—in units with low engagement (slope = 0.24, *p* < 0.001).

Consequently, the conditional indirect associations of breakroom culture with burnout via POS became progressively stronger as unit‐level team work engagement increased (see Table 2). The indices of moderated mediation were significant for both low well‐being (index = −0.06, 95% CI [–0.11, −0.03]) and disengagement (index = −0.05, 95% CI [–0.09, −0.02]), confirming that the strength of the indirect association depends on the unit’s team work engagement.

### 3.4. Robustness Checks

To assess the robustness of our hypothesised moderated mediation model, we compared its fit with several theoretically plausible alternative models. All alternative models exhibited an inferior fit to the hypothesised model (see Supporting Material 2, Tables S4–S5 for complete path coefficients and fit comparisons).

Additionally, we re‐estimated the full model after excluding the single unit with rwg(j) < 0.70 for breakroom culture; all path coefficients, indirect effects and the interaction term remained stable in direction, magnitude and statistical significance (Supporting Material 2, Table S2, Part B). Similarly, omitting all covariates from the model did not alter the pattern of results. These sensitivity analyses collectively strengthen confidence in the robustness of the proposed theoretical pathways.

## 4. Discussion

### 4.1. Interpretation of Findings and Theoretical Contributions

This multilevel study provides robust evidence that a supportive breakroom culture is negatively associated with nurse burnout through its positive association with POS—an informal‐organisational support pathway that is significantly amplified in units with a cohesive, engaged team‐level work engagement. The indirect associations via POS were statistically significant, while the direct associations of breakroom culture with both outcomes were nonsignificant in the present sample. This pattern indicates that POS accounts for the observed covariance between breakroom culture and burnout indicators, consistent with the hypothesised mediating pathway. We note that this does not preclude the existence of other unmeasured mediating mechanisms.

The primary theoretical contribution of this study is the identification of a context‐specific informal‐organisational support pathway. While the broader sequence of coworker support ⟶ POS ⟶ well‐being has established theoretical grounding [8, 13], our work extends this knowledge in two specific ways: (a) by demonstrating that informal, restorative breakroom interactions serve as a distinct and influential source of such support and (b) by identifying team‐level work engagement as a critical contextual amplifier of this process. The mediation finding advances OST [8, 31] by demonstrating that peers, through their informal supportive interactions in liminal workspaces, can act as symbolic agents of the organisation. Their behaviour provides cues that nurses use to form global judgements about organisational care, even in the absence of direct supervisory action [15, 32]. This mechanism may carry particular weight in contexts characterised by collectivism and high power distance—such as China—where informal relational networks (guanxi) serve as a fundamental social currency [19, 20]. In hierarchical healthcare systems, frontline nurses may perceive a distance from formal organisational authority; supportive peer interactions in the breakroom could therefore function as a critical, accessible proxy for organisational care. Positive breakroom culture, consequently, may be associated with partially compensating for perceived deficiencies in top‐down support, making the informal‐organisational support pathway a potentially vital, culturally resonant buffer against burnout. However, cross‐cultural comparative studies are needed to empirically verify whether this pathway is indeed stronger in such contexts than in more individualistic or low‐power‐distance settings. Cross‐cultural propositions for future testing are provided in Supporting Material 5.

The indirect association was slightly larger in magnitude for low psychological well‐being (*β* = −0.18) than for disengagement (*β* = −0.16). While overlapping confidence intervals preclude strong claims about differential effects, the pattern is theoretically consistent with a conservation of resources perspective [33] (see also [12]). From a conservation of resources perspective [33], low psychological well‐being represents a relatively immediate affective response to resource depletion and may be more readily associated with the socio‐emotional resources signalled through POS. Disengagement (cynicism), in contrast, reflects a deeper, more resilient cognitive‐attitudinal defence mechanism developed over time to cope with chronic stress. Reversing such entrenched detachment may require more sustained or intense supportive experiences than could be captured in a single cross‐sectional assessment. Future longitudinal research should examine whether the relative strength of these indirect effects shifts over the course of burnout development and recovery.

The significant moderating role of unit‐level team work engagement reveals a crucial contextual contingency. The positive association between breakroom culture and POS was strongest in units with high team work engagement (slope = 0.54), moderate in units with average engagement (slope = 0.39) and weakest—yet still significant—in units with low engagement (slope = 0.24). This contextual reinforcement effect indicates that when supportive acts in the liminal breakroom space are consistent with the shared experience of a positive, engaged team environment [17, 28], they are more likely to be interpreted as genuine, normative reflections of a caring organisational system. Conversely, in units with poor team work engagement, positive breakroom interactions may be perceived as isolated exceptions, weakening their impact on organisational judgements [18]. Importantly, the persistence of a significant, albeit attenuated, indirect effect even in low‐engagement units suggests that improving breakroom culture may be associated with benefits regardless of existing team dynamics. However, nurse managers should not view breakroom enhancements as a substitute for addressing fundamental deficits in team functioning; rather, improvements in informal spaces can serve as a complementary entry point for broader cultural change. This synergy between formal and informal social environments points to an integrated ecosystem for well‐being, where alignment between team work engagement and peer support creates mutually reinforcing, coherent signals of organisational support [31].

The indirect effects observed in this study, while modest in magnitude (*β* = −0.18 for low well‐being, −0.16 for disengagement), carry practical significance. On a five‐point scale, a one‐unit increase in breakroom culture corresponds to a 0.18‐unit reduction in low well‐being scores through enhanced POS. In units with a high team work engagement, this association strengthens to 0.24. Given the chronic nature of burnout and the difficulty of achieving large effect sizes in organisational interventions, even small improvements at scale can translate into meaningful reductions in burnout prevalence and associated turnover intentions [2]. From a population health perspective, such incremental gains, when multiplied across hundreds of nursing units, have the potential to substantially improve workforce sustainability and patient care quality.

### 4.2. Implications for Nursing Management

The findings translate into concrete, actionable strategies for nurse leaders. These strategies are summarised in Box 1 and are designed to be feasible across diverse clinical settings, including those with limited budgets and high turnover.

First, nurse managers should reconceptualise breakrooms as strategic, recovery‐oriented sanctuaries rather than merely utilitarian spaces. Evidence‐based environmental enhancements—such as creating a designated quiet zone, introducing comfortable seating, improving natural light, and displaying positive message walls—can foster a supportive culture with minimal financial outlay [6, 14]. We recommend that managers conduct a simple annual audit of breakroom physical and psychosocial conditions using a brief checklist (see Supporting Material 4 for a prototype).

Second, relational leadership behaviours that model and nurture positive breakroom norms should be explicitly cultivated. Authentic leadership theory emphasises transparency, balanced processing, and relational authenticity [34]; these behaviours can be demonstrated by managers being present in the breakroom regularly, particularly during peak break times, listening to staff concerns and offering genuine empathy. Such visible presence signals that peer support and recovery are organisationally valued. Leadership development programmes should include modules on building and sustaining supportive informal subcultures, framing these as core competencies for staff well‐being and retention [16, 34].

Third, written task guidelines should be developed and disseminated to explicitly prohibit noneducational, nonclinical chores from encroaching on break time. Our findings indicate that even registered nurses may experience role ambiguity and unwarranted tasks during breaks. Clear policies that protect break time as uninterrupted recovery time, coupled with regular audits by unit managers, can strengthen the signal that the organisation genuinely cares about employee well‐being, thereby enhancing POS [15]. Such structural reforms address the root causes of burnout rather than merely its symptoms. Research on hospital work‐hour policies has demonstrated that organisational regulations limiting extended work periods can reduce fatigue‐related errors and improve nurse well‐being [35], illustrating how institutional policies that safeguard recovery time can serve as tangible expressions of organisational support.

Fourth, routine monitoring of unit‐level team work engagement should be embedded into quality improvement cycles. The UWES‐9‐T is brief, freely available, and sensitive to change [17]. We recommend administering it biannually and reviewing aggregated results with staff within 1 month. Transparent discussion of team work engagement data enables shared diagnosis of strengths and deficits and empowers teams to co‐design corrective actions. This participatory approach aligns with principles of organisational learning and psychological safety [28].

Fifth, interventions to strengthen team cohesion should be pursued synergistically with breakroom enhancements. Regular team‐building activities that extend positive interactions from the breakroom into formal work processes—such as peer recognition during handovers, collaborative problem‐solving huddles and shared celebration of achievements—can create a coherent, reinforcing social environment [36, 37]. In units where intensive team‐building is not feasible, even brief structured peer‐reflection sessions conducted via mobile platforms (e.g., WeChat groups) have demonstrated acceptability and feasibility [36, 37].

Crucially, these recommendations are stratified by leadership level to match decision‐making authority and resource availability. For frontline nurse managers, the emphasis is on relational presence, role modelling and low‐cost environmental tweaks. For nursing directors, we recommend allocating a dedicated budget for breakroom refurbishment and including team work engagement metrics in organisational dashboards. For hospital administrators and policy makers, embedding staff relational well‐being into accreditation standards and strategic workforce plans would signal institutional commitment and enable sustainable funding. This tiered approach responds directly to the feasibility concerns raised by clinical managers and enhances the translational value of our findings.

**Box 1: Practical strategies for nurse managers.**
(1) Be present in the breakroom regularly and informally, particularly during peak break periods, to listen, offer empathy and model open communication.
(2) Recognize peer support publicly. Identify observed acts of collegial support and acknowledge them during shift handovers or on a visible recognition board.
(3) Upgrade the physical environment with low‐cost changes. Create a “positive message wall” for anonymous notes of gratitude or encouragement. Designate a quiet corner with comfortable seating and soft lighting for psychological detachment.
(4) Embed breakroom norms into formal team routines by briefly sharing positive examples of peer support during regular team meetings. Include “collegial support in informal settings” as a criterion for monthly team awards or annual performance feedback.
(5) Strengthen team cohesion synergistically. Conduct regular team‐building activities that extend positive interactions from the breakroom into formal work processes. Administer the UWES‐9‐T biannually and review unit‐level results with staff within 1 month to monitor engagement and address deficits early.
(6) Match interventions to leadership level.
 Nurse managers: Focus on relational presence, role modelling, and local environmental enhancements.
 Nursing directors: Allocate dedicated budget for breakroom refurbishment and include team work engagement metrics in quality dashboards.
 Hospital administrators: Embed staff relational well‐being into accreditation standards and strategic workforce plans.

### 4.3. Implications for Patient Care and Safety

Although this study did not directly measure patient outcomes, the well‐established association between nurse burnout and care quality provides a rationale for extrapolating our findings to patient care [2, 3]. Low psychological well‐being and disengagement among nurses have been consistently linked to increased risk of medication errors, lower patient satisfaction, reduced empathy and even elevated mortality rates [2, 15]. Our results suggest that a supportive breakroom culture may be indirectly associated with better patient outcomes through its links to reduced burnout. However, this pathway remains speculative until directly tested using linked nurse–patient data, as patient outcomes were not measured in the present study. Recent evidence confirms that POS is directly associated with positive nurse work attitudes and reduced moral distress [38] and that positive team work engagement reduces nursing care rationing—a key behavioural pathway linking nurse well‐being to missed care and patient complications [12, 16, 39]. Extending this logic, our findings suggest that breakroom culture, as an antecedent of POS and a synergistic partner of team work engagement, may be indirectly associated with safer, more compassionate care. Nurse managers should therefore consider investments in breakroom enhancement and team work engagement not as discretionary amenities but as components of a comprehensive patient safety strategy. Future research should formally test this extended chain (breakroom culture ⟶ POS ⟶ burnout ⟶ patient indicators) using linked nurse–patient data and objective quality metrics.

### 4.4. Limitations and Future Research Directions

Several limitations should be considered when interpreting these findings. First, the cross‐sectional design precludes definitive causal conclusions. Although we employed robust theoretical grounding, latent variable decomposition, and tested reverse causality models that demonstrated poorer fit, causal claims regarding the direction of effects between breakroom culture, POS, and burnout cannot be conclusively established. Longitudinal or quasiexperimental intervention studies are urgently needed to confirm temporal dynamics and treatment efficacy [40]. We encourage researchers to employ repeated‐measures designs with at least three time points to enable rigorous tests of mediation over time.

Second, although we implemented procedural remedies to mitigate common method variance [22], all data were self‐reported, raising the possibility of inflated associations due to shared method variance. Future studies should incorporate multisource data, such as peer ratings of breakroom culture, supervisor assessments of team work engagement and objective turnover or absenteeism records. Experience sampling methods could also capture real‐time fluctuations in breakroom interactions and momentary well‐being [40].

Third, our operationalisation of team‐level work engagement using the UWES‐9‐T, while psychometrically sound, does not capture the full multidimensionality of team climate as conceptualised by instruments such as the TCI [28]. Team‐level work engagement shares conceptual overlap with the affective‐motivational dimension of team climate but is not interchangeable with the broader climate construct. We recommend that future studies replicate our model using the TCI or other comprehensive climate measures to establish convergent validity and determine whether specific climate dimensions are particularly influential in moderating the breakroom culture–POS relationship. To facilitate such replication, information on obtaining the full correlation and covariance matrices is provided in Supporting Material 3.

Fourth, the WHO‐5 was not designed as an occupational exhaustion measure. While reverse‐scored WHO‐5 correlates strongly with MBI‐GS emotional exhaustion (*r* = −0.73 in our validation subsample) and yields an indirect effect pattern comparable to that of the MBI‐GS (Δab = −0.02, 95% CI [–0.07, 0.04]; see Supporting Material 3), they are not equivalent. We have relabelled this variable as “low psychological well‐being (exhaustion‐related distress)” to reflect this distinction transparently. However, this choice entails a trade‐off: our findings for low well‐being are not directly comparable with studies using burnout‐specific measures, and the construct coverage differs from that of established burnout instruments. Future research should replicate our findings using validated burnout‐specific instruments such as the Maslach Burnout Inventory or Oldenburg Burnout Inventory [41] to determine whether the informal‐organisational support pathway operates with comparable magnitude across different health indicators and to enable direct meta‐analytic integration with the broader burnout literature.

Fifth, although we controlled for key individual‐level covariates and conducted supporting analyses showing that shift pattern did not moderate the hypothesised paths, our model did not include important unit‐level confounders such as patient‐to‐nurse ratios, average workload or specific unit leadership styles. These factors could independently influence both team work engagement and burnout levels. Future multilevel studies should incorporate such objective or aggregated unit‐level measures to obtain more precise estimates of the contextual effects of team work engagement and to rule out alternative explanations for the shared variance observed at the unit level.

Sixth, generalisability may be constrained as data were collected from urban tertiary hospitals in one region of China using a convenience sampling strategy. Although our sample was diverse in unit types and shift patterns, the convenience sampling approach may have introduced selection bias, as units with more supportive management may have been more willing to participate. The findings may not directly transfer to other hospital tiers (e.g., secondary or community hospitals), rural settings or different cultural contexts. Stratified random sampling across hospital levels and regions is recommended for future research to enhance generalisability. Cross‐cultural comparative research is explicitly called for to test the moderating role of societal culture on the informal‐organisational support pathway, as articulated in Section 4.1 and detailed in Supporting Material 5.

Seventh, our measure of breakroom culture, while rigorously developed and contextually validated, focused exclusively on positive, supportive interactions. Emerging evidence suggests that negative workplace behaviours—such as incivility, ostracism and interpersonal conflict—may exert stronger, more direct effects on burnout that operate independently of POS [40, 42]. Future studies should employ dual‐factor measurement approaches that capture both positive and negative dimensions of breakroom interactions. Adapting the Workplace Incivility Scale [40] or the Workplace Ostracism Scale [42] to the breakroom context would enable examination of whether negative dynamics have incremental predictive validity beyond the supportive culture measured here.

Eighth, intervention research is critically needed to translate our correlational findings into evidence‐based management practice. We have proposed a tiered intervention framework (Box 1) that now requires rigorous evaluation through cluster randomised controlled trials or stepped‐wedge designs. Such studies should assess not only burnout and POS but also implementation outcomes (acceptability, fidelity and cost) and patient‐level endpoints. Participatory approaches that engage nurses and managers in co‐designing interventions are likely to enhance contextual fit and sustainability [9].

Summary of key limitations. Readers are cautioned to consider the following constraints when interpreting these findings: (a) the cross‐sectional design precludes causal inference; (b) the WHO‐5 measures psychological well‐being, not occupational exhaustion per se, and our relabelling of this variable as “low psychological well‐being (exhaustion‐related distress)” is a proxy operationalisation. Supporting validation using the MBI‐GS (Supporting Material 3) supports the robustness of the indirect effect pattern; (c) team‐level work engagement (UWES‐9‐T) captures only the affective‐motivational dimension of team climate; (d) the breakroom culture scale is study‐specific and requires independent validation; (e) convenience sampling and single‐region data collection limit generalisability and (f) all data were self‐reported.

## 5. Conclusion

This multilevel study demonstrates that a supportive breakroom culture is negatively associated with nurse burnout through enhanced POS—an informal organisational support pathway amplified by positive team‐level work engagement. The findings reposition breakroom quality as a strategic lever for workforce resilience. An integrated leadership approach that cultivates restorative peer spaces, authentic relational presence and cohesive team environments offers a sustainable pathway to mitigate burnout through aligning formal structures with informal social support systems.

## Author Contributions

Yanjun Wu: conceptualization, investigation, and writing–original draft preparation.

Yanyan Lu: methodology, data curation, and formal analysis.

Wubin He: supervision, project administration, funding acquisition, and writing–review and editing.

## Funding

This study received funding from the Horizontal Project, Jinzhou Medical University (A2024007).

## Ethics Statement

Approval for this study was obtained from the Ethics Committee of Jinzhou Medical University (Approval No: JZMULL2025328).

All participants provided informed consent prior to their involvement in the study.

## Conflicts of Interest

The authors declare no conflicts of interest.

## Supporting Information

Additional supporting information can be found online in the Supporting Information section.

The following supporting materials are available with the online version of this article:

## Supporting information


**Supporting Information 1** Supporting Material 1. Measurement instruments (English and Mandarin Chinese versions). Complete item lists for all scales used in the study, including the Breakroom Culture Scale, Perceived Organisational Support Scale, WHO‐5 Well‐Being Index, Disengagement (Cynicism) Subscale, UWES‐9‐T Team Version, and General Perceived Coworker Support Scale. Supporting Material 2. Additional analyses and technical details. Sample characteristics (Table S3), breakroom culture scale psychometric properties (Table S1), multilevel confirmatory factor analysis results (Table S2), aggregation statistics and sensitivity analysis (Table S2, Part B), reverse mediation model results (Table S4), alternative model comparisons (Table S5), Mplus syntax for the hypothesised cross‐level moderated mediation model, and extended data and code availability statement. Supporting Material 3. Extended theoretical propositions and supplementary analyses. MBI‐GS supplementary validation comparing indirect effect patterns using WHO‐5 versus the MBI‐GS emotional exhaustion subscale (Section A), shift pattern supplementary moderation analyses (Section B), sensitivity analysis excluding covariates (Section C) and de‐identified correlation and covariance matrix availability (Section D). Supporting Material 4. Prototype breakroom audit checklist for nurse managers. A practical, adaptable tool for annual assessment of breakroom physical and psychosocial conditions. Supporting Material 5. Cross‐cultural hypotheses for future research. Formal cross‐cultural research propositions and suggested multination design for testing the generalisability of the informal‐organisational support pathway.

**Supporting Information 2** STROBE Checklist

## Data Availability

The raw datasets generated and analysed during the current study are not publicly available due to institutional restrictions and the sensitive nature of personnel well‐being data. The complete Mplus syntax used for the main analyses and all supporting models is provided in Supporting Material 2. De‐identified within‐ and between‐level correlation and covariance matrices sufficient to reproduce the structural model results are available from the corresponding author upon reasonable request and with approval from the participating hospitals’ ethics committees. Additionally, a synthetic dataset with identical statistical properties (means, standard deviations and intercorrelations) is available from the corresponding author upon reasonable request and subject to the same ethical approval.
