# Supplementary material for: Breakroom Culture and Nurse Burnout: A Multilevel Moderated Mediation Analysis
Source: J Nurs Manag. 2026 Aug 21;2026:5545718. doi: 10.1155/jonm/5545718 (PMC13498857; doi:10.1155/jonm/5545718)
Supplement: Supplementary file 1 [file JONM-2026-5545718-s001.docx]

| **Section** | **Item No** | **Recommendation** | **Reported on Page(s)** | **Remarks** |
| --- | --- | --- | --- | --- |
| Title and abstract |  |  |  |  |
|  | 1 | (a) Indicate the study's design with a commonly used term in the title or the abstract | Title; Abstract | Title uses "Multilevel Moderated Mediation Analysis"; Abstract explicitly states "cross-sectional multilevel study" |
|  |  | (b) Provide in the abstract an informative and balanced summary of what was done and what was found | Abstract | Includes Aim, Background, Methods, Results, Conclusion, and Implications |
| Introduction |  |  |  |  |
| Background/rationale | 2 | Explain the scientific background and rationale for the investigation being reported | Introduction, ¶1–4 | JD-R theory and OST integration; research gap; Chinese healthcare contextualization |
| Objectives | 3 | State specific objectives, including any prespecified hypotheses | Introduction, final ¶ | Three aims; H1, H2, H3a, H3b formally listed |
| Methods |  |  |  |  |
| Study design | 4 | Present key elements of study design early in the paper | Methods 2.1 | Cross-sectional multilevel design; nurses nested within clinical units |
| Setting | 5 | Describe the setting, locations, and relevant dates | Methods 2.1 | March–October 2025; three tertiary public hospitals in Liaoning Province, China |
| Participants | 6 | (a) Give the eligibility criteria, and the sources and methods of selection | Methods 2.1 | Full-time registered nurses in 36 clinical units; convenience sampling |
| Variables | 7 | Clearly define all outcomes, exposures, predictors, potential confounders, and effect modifiers | Methods 2.3 | All variables defined with source citations, response formats, and reliability coefficients |
| Data sources/ measurement | 8* | For each variable, give sources of data and details of methods of assessment | Methods 2.3; Supplementary Material 1 | Scales described with full psychometric details; six-step validation for breakroom culture scale |
| Bias | 9 | Describe any efforts to address potential sources of bias | Methods 2.3; Methods 2.4; Discussion 4.4 | Procedural remedies for common method bias; FIML for missing data; robustness checks |
| Study size | 10 | Explain how the study size was arrived at | Methods 2.1 | 720 nurses from 36 units; mean cluster size = 20; adequate for multilevel modelling |
| Quantitative variables | 11 | Explain how quantitative variables were handled in the analyses | Methods 2.3; Methods 2.4 | Continuous variables used; shift pattern dichotomized; latent variable decomposition |
| Statistical methods | 12 | (a) Describe all statistical methods, including those used to control for confounding | Methods 2.4 | MSEM with MLR; FIML; latent decomposition; bootstrap 5,000; covariates in all models |
|  |  | (b) Describe any methods used to examine subgroups and interactions | Methods 2.3; Supplementary Material 3 | Cross-level interaction; conditional indirect effects at ±1 SD; shift pattern moderation |
|  |  | (c) Explain how missing data were addressed | Methods 2.4 | Missing data < 2%; FIML under MAR assumption |
|  |  | (d) Describe analytical methods taking account of sampling strategy | Methods 2.4 | Multilevel SEM for nested data; ICCs; TYPE = TWOLEVEL RANDOM in Mplus |
|  |  | (e) Describe any sensitivity analyses | Results 3.4; Supplementary Material 2–3 | Five alternative models; low-rwg unit exclusion; covariate omission |
| Results |  |  |  |  |
| Participants | 13* | (a) Report numbers of individuals at each stage of study | Methods 2.1; Results 3.1 | 720 nurses from 36 units; detailed demographics in SM2 Table S3 |
|  |  | (b) Give reasons for non-participation at each stage | Not reported | Convenience sampling; non-response rate not explicitly stated |
|  |  | (c) Consider use of a flow diagram | Not included | No flow diagram |
| Descriptive data | 14* | (a) Give characteristics of study participants and information on exposures and potential confounders | Results 3.1; Table 1; SM2 Table S3 | 92.5% female; mean age 32.4 years (SD = 7.1); 74% rotating shifts; 36 units |
|  |  | (b) Indicate number of participants with missing data for each variable | Methods 2.4 | Missing data < 2% overall |
| Outcome data | 15* | Report numbers of outcome events or summary measures | Results 3.1; Table 1 | Means, SDs, ICCs, and intercorrelations reported |
| Main results | 16 | (a) Give unadjusted and confounder-adjusted estimates with precision (95% CI) | Results 3.3; Table 2 | Adjusted estimates reported with 95% BCa CIs; covariates listed |
|  |  | (b) Report category boundaries when continuous variables were categorized | Not applicable | Continuous variables used; TWE split at ±1 SD for simple slopes |
|  |  | (c) Consider translating estimates of relative risk into absolute risk | Not applicable | Study reports standardized path coefficients and indirect effects |
| Other analyses | 17 | Report other analyses—subgroups, interactions, sensitivity analyses | Results 3.3; Results 3.4; SM3 | Cross-level interaction; conditional indirect effects; moderated mediation indices; alternative models; shift pattern moderation |
| Discussion |  |  |  |  |
| Key results | 18 | Summarise key results with reference to study objectives | Discussion 4.1, ¶1 | Informal-organisational support pathway supported; team work engagement as amplifier |
| Limitations | 19 | Discuss limitations, taking into account sources of potential bias or imprecision | Discussion 4.4; Summary of key limitations | Eight limitations; summary box with six key constraints |
| Interpretation | 20 | Give a cautious overall interpretation considering objectives, limitations, and other relevant evidence | Discussion 4.1; Discussion 4.3; Conclusion | Associational language throughout; patient outcome pathway stated as "speculative" |
| Generalisability | 21 | Discuss the generalisability (external validity) of the study results | Discussion 4.4, ¶6 | Urban tertiary hospitals in one region of China; convenience sampling; recommendations for cross-cultural replication |
| Other information |  |  |  |  |
| Funding | 22 | Give the source of funding and the role of the funders | Funding statement | Horizontal Project, Jinzhou Medical University (A2024007) |
| Ethics approval | — | State that ethical approval was obtained and provide approval number | Methods 2.2; Ethics approval section | Jinzhou Medical University Ethics Committee (Approval No: JZMULL2025328); written informed consent obtained |
