# Supplementary material for: Breakroom Culture and Nurse Burnout: A Multilevel Moderated Mediation Analysis
Source: J Nurs Manag. 2026 Aug 21;2026:5545718. doi: 10.1155/jonm/5545718 (PMC13498857; doi:10.1155/jonm/5545718)
Supplement: Supplementary file 2 [file JONM-2026-5545718-s002.docx]

**SUPPLEMENTARY MATERIALS**

**Table of Contents
Supplementary Material 1 – Measurement Instruments
Supplementary Material 2 – Additional Analyses and Technical Details
Supplementary Material 3 – Extended Theoretical Propositions and
Supplementary Analyses
Supplementary Material 4 – Prototype Breakroom Audit Checklist
Supplementary Material 5 – Cross-Cultural Hypotheses for Future Research**

**Supplementary Material 1 – Measurement Instruments (English and Mandarin Chinese Versions)**
Description: Complete item lists for all scales used in the study, presented in both the original (or source) English form and the adapted Mandarin Chinese version administered to participants. All scales are either in the public domain, freely available for non‑commercial scientific research, or used with permission. Permissions have been archived and can be provided upon request.

**1. Breakroom Culture Scale**

Source: Developed contextually for this study based on the theoretical construct of perceived coworker support (Chiaburu & Harrison, 2008). Breakroom culture is defined as the shared perception of supportive, caring, and collegial peer interactions occurring specifically within the staff breakroom during rest periods—a context-specific manifestation of informal coworker support (Chiaburu & Harrison, 2008) grounded in social exchange and organisational support theory (Eisenberger et al., 1986). The scale was developed through a systematic six-step validation process (see Supplementary Material 2, Table S1 for full psychometric details).

**Response format:** 5‑point Likert scale (1 = Strongly disagree, 5 = Strongly agree).

**Reliability in this study:** Cronbach's α = 0.89; CR = 0.90; AVE = 0.64.
**Note:** As a study-specific, contextually validated measure, independent validation in future research is encouraged.

| **Item** | **English Item** | **Mandarin Chinese Item** |
| --- | --- | --- |
| BC1 | I can count on my colleagues for a supportive talk when we are in the breakroom. | 在休息室里，我可以依靠同事进行支持性的交谈。 |
| BC2 | In the breakroom, my colleagues and I genuinely care about each other’s well‑being. | 在休息室里，我和同事们真诚地关心彼此的福祉。 |
| BC3 | The breakroom is a place where I feel comfortable sharing work‑related difficulties. | 休息室是一个让我感到可以自在分享工作困难的地方。 |
| BC4 | My colleagues offer encouragement to me during break time. | 同事们在休息时间会给予我鼓励。 |
| BC5 | Overall, the atmosphere in our breakroom is warm and supportive. | 总的来说，我们休息室的氛围是温暖和支持性的。 |

**2. Perceived Organizational Support (POS) Scale – Revised Public‑Domain Version**

**Source:** Eisenberger, R., Shanock, L. R., & Wen, X. (2020). Perceived organizational support: Why caring about employees counts. Annual Review of Organizational Psychology and Organizational Behavior, 7, 101–124. Public‑domain version, freely available for use without permission.

**Response format:** 5‑point Likert scale (1 = Strongly disagree, 5 = Strongly agree).

Reliability in this study: Cronbach’s α = 0.92.

| **Item** | **English Item** | **Mandarin Chinese Item** |
| --- | --- | --- |
| POS1 | The organization strongly considers my goals and values. | 组织非常重视我的目标和价值观。 |
| POS2 | Help is available from the organization when I have a problem. | 当我遇到问题时，可以从组织获得帮助。 |
| POS3 | The organization would forgive an honest mistake on my part. | 组织会原谅我无意中犯的错误。 |
| POS4 | The organization really cares about my well‑being. | 组织真正关心我的福祉。 |
| POS5 | The organization shows very little concern for me. (R) | 组织对我几乎不关心。（反向计分） |
| POS6 | The organization is willing to help me when I need a special favor. | 当我需要特殊帮助时，组织愿意提供支持。 |
| POS7 | The organization cares about my opinions. | 组织在意我的意见。 |
| POS8 | The organization takes pride in my accomplishments at work. | 组织为我工作中的成就感到自豪。 |

(R) = reverse‑scored item.

**3. Outcome Measures**

**3a. Low Psychological Well-being (Reverse-Scored WHO-5 Well-Being Index)**

**Source:** Topp, C. W., Østergaard, S. D., Søndergaard, S., & Bech, P. (2015). The WHO-5 Well-Being Index: A systematic review of the literature. Psychotherapy and Psychosomatics, 84(3), 167–176. Freely available for use without permission.

**Note:** The WHO-5 Well-Being Index was developed to measure subjective psychological well-being, not occupational exhaustion per se. In this study, the scale is reverse-scored and labelled as 'low psychological well-being' to accurately reflect what the instrument measures. Low psychological well-being is positioned as a standalone health indicator capturing the erosion of positive mental health (diminished vitality, reduced positive affect, and loss of interest), which is conceptually distinct from but empirically associated with burnout dimensions. Convergent validity with the emotional exhaustion subscale of the Maslach Burnout Inventory-General Survey (MBI-GS; Maslach et al., 1996) was r = –0.73 (*P* < 0.001) in a separate validation subsample (N = 120). The MBI-GS was selected for this correlational validation as the most widely recognised international standard for burnout assessment. The MBI-GS is a copyrighted instrument (Mind Garden, Inc.); its items are not reproduced in this manuscript or its supplementary materials. Additionally, supplementary mediation analysis on this same subsample confirmed that the indirect effect pattern (breakroom culture → POS → outcome) is comparable when the outcome is operationalised using reverse-scored WHO-5 versus the MBI-GS emotional exhaustion subscale (Δab = –0.02, 95% CI [–0.07, 0.04]; see Supplementary Material 3 for full details). These convergent findings support the sensitivity of the WHO-5 to exhaustion-related distress while acknowledging it measures the broader construct of psychological well-being. For a full discussion, see the Methods section (Main Text 2.3) and Limitations (4.4)."

**Response format:** 6‑point frequency scale (0 = At no time, 5 = All of the time).
**Note:** Items are reverse‑scored so that lower scores indicate lower psychological well-being/higher exhaustion-related distress.

Reliability in this study: Cronbach’s α = 0.85.

| **Item** | **English Item (past two weeks)** | **Mandarin Chinese Item** |
| --- | --- | --- |
| WHO1 | I have felt cheerful and in good spirits. (R) | 我感到快乐、心情舒畅。（反向计分） |
| WHO2 | I have felt calm and relaxed. (R) | 我感到平静和放松。（反向计分） |
| WHO3 | I have felt active and vigorous. (R) | 我感到充满活力和精力充沛。（反向计分） |
| WHO4 | I woke up feeling fresh and rested. (R) | 我醒来时感到神清气爽、休息充分。（反向计分） |
| WHO5 | My daily life has been filled with things that interest me. (R) | 我的日常生活中充满了令我感兴趣的事情。（反向计分） |

(R) = reverse‑scored item.

**3b. Disengagement (Cynicism) Subscale**

**Source:** Adapted from publicly available burnout research literature; conceptually based on the Copenhagen Burnout Inventory (Kristensen et al., 2005). Freely available for academic use.

**Response format:** 5-point Likert scale (1 = Strongly disagree, 5 = Strongly agree).

**Reliability in this study:** Cronbach's α = 0.82.

| **Item** | **English Item** | **Mandarin Chinese Item** |
| --- | --- | --- |
| DIS1 | I have become more cynical about whether my work contributes anything. | 我对我工作是否有贡献变得更加怀疑。 |
| DIS2 | I doubt the significance of my work. | 我怀疑自己工作的意义。 |
| DIS3 | I have lost interest in my work. | 我对工作失去了兴趣。 |
| DIS4 | I feel that my work is meaningless. | 我觉得我的工作毫无意义。 |

**4. Team-Level Work Engagement – Team Version of the Utrecht Work Engagement Scale (UWES-9-T)**

Source: Schaufeli, W. B., Bakker, A. B., & Salanova, M. (2006). The measurement of work engagement with a short questionnaire: A cross-national study. Educational and Psychological Measurement, 66(4), 701–716. Used in accordance with its availability terms for non-commercial scientific research.

**Note:** In this study, the UWES-9-T is used to measure team-level work engagement—the shared, collective experience of vigour, dedication, and absorption within a work team—operationalised through a reference-shift composition model (Klein & Kozlowski, 2000). This focused measurement strategy was selected for three reasons: (a) the UWES-9-T's vigour-dedication-absorption dimensions directly capture the affective-motivational core relevant to the hypothesised moderation mechanism; (b) the instrument has been extensively validated in Chinese healthcare populations; and (c) its brevity (9 items) reduces respondent burden. It is not used as a measure of the multidimensional construct of team climate as assessed by instruments such as the Team Climate Inventory (TCI; Anderson & West, 1998). Readers are referred to the Methods section (Main Text 2.3) for a detailed rationale and the Limitations section (4.4) for a discussion of this distinction.

**Response format:** 7‑point frequency scale (0 = Never, 6 = Always).
**Instruction:** Participants rated their team using the stem: “As a team, we…”

**Reliability (aggregated) in this study:** Cronbach's α = 0.93.

| **Item** | **English Item (with stem)** | **Mandarin Chinese Item** |
| --- | --- | --- |
| T1 | As a team, we are enthusiastic about our jobs. | 作为我们的团队，我们对工作充满热情。 |
| T2 | As a team, our job inspires us. | 作为我们的团队，工作激励着我们。 |
| T3 | As a team, we are proud of the work that we do. | 作为我们的团队，我们为自己所做的工作感到自豪。 |
| T4 | As a team, we feel happy when we are working intensely. | 作为我们的团队，当投入工作时我们感到快乐。 |
| T5 | As a team, we are immersed in our work. | 作为我们的团队，我们沉浸在工作中。 |
| T6 | As a team, we are energetic and resilient in our work. | 作为我们的团队，我们在工作中充满活力且坚韧不拔。 |
| T7 | As a team, we persevere even when things do not go well. | 作为我们的团队，即使事情进展不顺利，我们也能坚持。 |
| T8 | As a team, we can continue working for very long periods. | 作为我们的团队，我们可以持续工作很长时间。 |
| T9 | As a team, we are dedicated to our work. | 作为我们的团队，我们全身心投入工作。 |

**5. General Perceived Coworker Support Scale (used for discriminant validity testing only)**

**Source:** Chiaburu, D. S., & Harrison, D. A. (2008). Do peers make the place? Conceptual synthesis and meta-analysis of coworker effects on perceptions, attitudes, OCBs, and performance. Journal of Applied Psychology, 93(5), 1082–1103.

**Response format:** 5-point Likert scale (1 = Strongly disagree, 5 = Strongly agree).

| **Item** | **English Item** | **Mandarin Chinese Item** |
| --- | --- | --- |
| GCS1 | My coworkers are willing to listen to my work‑related problems. | 同事愿意倾听我工作中遇到的问题。 |
| GCS2 | My coworkers help me with difficult assignments. | 同事会帮助我完成困难的任务。 |
| GCS3 | My coworkers care about my well‑being. | 同事关心我的福祉。 |
| GCS4 | My coworkers provide me with support and encouragement. | 同事给予我支持与鼓励。 |

**Supplementary Material 2 – Additional Analyses and Technical Details**
**Description:** Detailed sample characteristics, multilevel confirmatory factor analysis, justification for aggregation, full psychometric properties of the newly developed Breakroom Culture Scale, Mplus syntax for the hypothesised model, robustness checks, and extended data/code availability statement. All content is presented in English.

**Table S1. Psychometric Properties of the Breakroom Culture Scale (Development and Validation)**

| **Phase / Property** | **Indicator / Criterion** | **Result** |
| --- | --- | --- |
| **Step 1: Item generation** | Number of items generated | 12 |
| **Step 2: Content validity** | Expert panel size | 5 |
|  | Expert panel composition | Two nursing managers (>15 yrs experience), two senior clinical nurses (>10 yrs), one occupational health psychologist |
|  | Item retention criterion | I-CVI ≥ 0.78 |
|  | Items deleted (I-CVI < 0.78) | 4 |
|  | Scale-level CVI (S-CVI/Ave) | 0.94 |
| **Step 3: Exploratory factor analysis (pilot sample, N = 180)** | Sample size (independent pilot) | 180 |
|  | KMO | 0.87 |
|  | Bartlett's test | χ²(28) = 623.41, P < 0.001 |
|  | Eigenvalues (first factor) | 4.78 |
|  | Variance explained (first factor) | 68.4% |
|  | Factor loadings range | 0.72–0.89 |
|  | Items deleted (low/cross loading) | 3 |
| **Step 4: Confirmatory factor analysis (main sample, N = 720)** | Final scale items retained | 5 |
|  | CFI | 0.99 |
|  | RMSEA | 0.03 |
|  | SRMR | 0.02 |
| **Step 5: Discriminant validity (main sample, N = 720)** | Correlation with general coworker support | *r* = 0.51, P < 0.001 |
|  | Two-factor model (BrC + GCS) | CFI = 0.98, RMSEA = 0.04 |
|  | One-factor model (merged) | CFI = 0.84, RMSEA = 0.11 |
|  | Δχ² (df = 1) | 47.2, P < 0.001 |
|  | √AVE (BrC) vs. *r*(BrC, GCS) | 0.80 > 0.51 |
| **Step 6: Reliability** | Cronbach's α (main sample) | 0.89 |
|  | Composite reliability (CR) | 0.90 |
|  | Average variance extracted (AVE) | 0.64 |

Note: BrC = Breakroom Culture; GCS = General Coworker Support; I-CVI = Item-level Content Validity Index; S-CVI/Ave = Scale-level Content Validity Index (average). Discriminant validity was established through chi-square difference testing and the Fornell-Larcker criterion. The scale was developed contextually for this study; independent validation in future research is encouraged.

**Table S2, Part A. Standardised Factor Loadings from Multilevel Confirmatory Factor Analysis (Four-Factor Model)**

| **Scale / Item** | **Within-Level Loading** | **Between-Level Loading** |
| --- | --- | --- |
| **Breakroom Culture** |  |  |
| BC1 | 0.82 | 0.85 |
| BC2 | 0.85 | 0.88 |
| BC3 | 0.81 | 0.83 |
| BC4 | 0.78 | 0.80 |
| BC5 | 0.76 | 0.79 |
| CR / AVE | 0.90 / 0.65 | 0.92 / 0.70 |
| **POS** |  |  |
| POS1 | 0.85 | 0.87 |
| POS2 | 0.82 | 0.85 |
| POS3 | 0.78 | 0.81 |
| POS4 | 0.88 | 0.91 |
| POS5 (R) | 0.71 | 0.75 |
| POS6 | 0.83 | 0.86 |
| POS7 | 0.80 | 0.84 |
| POS8 | 0.86 | 0.89 |
| CR / AVE | 0.93 / 0.66 | 0.94 / 0.71 |
| **Low Psychological Well-being (WHO-5-R)** |  |  |
| WHO1 | 0.79 | — |
| WHO2 | 0.76 | — |
| WHO3 | 0.82 | — |
| WHO4 | 0.75 | — |
| WHO5 | 0.71 | — |
| CR / AVE | 0.86 / 0.55 | — |
| **Disengagement** |  |  |
| DIS1 | 0.80 | — |
| DIS2 | 0.77 | — |
| DIS3 | 0.74 | — |
| DIS4 | 0.69 | — |
| CR / AVE | 0.83 / 0.55 | — |

Note: The hypothesised four-factor model (Breakroom Culture, POS, Low Psychological Well-being, Disengagement) demonstrated excellent fit: CFI = 0.97, TLI = 0.96, RMSEA = 0.04, SRMRwithin = 0.03, SRMRbetween = 0.05. All loadings significant at *P* < 0.001. CR = composite reliability; AVE = average variance extracted; (R) = reverse-scored. Between-level loadings are not applicable for individual-level burnout dimensions due to insufficient between-unit variance for item-level decomposition.

**Part B. Aggregation Statistics and Sensitivity Analysis for Unit‑Level Constructs**

| Construct | Mean rwg(j) | rwg(j)range | Units with rwg(j)< 0.70 | ICC(1) | ICC(2) | Between‑level α | Sensitivity analysis (exclusion of low‑rwg unit) |
| --- | --- | --- | --- | --- | --- | --- | --- |
| Breakroom Culture | 0.82 | 0.68 – 0.92 | 1 | 0.38*** | 0.78 | 0.91 | No substantive change in path coefficients or indirect effects |
| Team-Level Work Engagement | 0.88 | 0.72 – 0.96 | 0 | 0.40*** | 0.82 | 0.93 | Not applicable |

Note. rwg(j) = within‑group agreement index for multi‑item scales; ICC(1) = proportion of variance between units; ICC(2) = reliability of unit means. Sensitivity analysis was performed by re-estimating the full moderated mediation model after removing the single unit with rwg(j) < 0.70 for Breakroom Culture. All parameter estimates remained stable in direction, magnitude, and statistical significance. *** *P* < 0.001.

**Table S3. Complete Demographic and Work Characteristics of the Sample (N = 720)**

| **Characteristic** | **Category** | **n** | **%** | **M** | **SD** |
| --- | --- | --- | --- | --- | --- |
| Gender | Female | 666 | 92.5 |  |  |
|  | Male | 54 | 7.5 |  |  |
| Age (years) |  |  |  | 32.4 | 7.1 |
| Education | Associate degree or below | 245 | 34.0 |  |  |
|  | Bachelor's degree | 451 | 62.6 |  |  |
|  | Master's degree or above | 24 | 3.4 |  |  |
| Total nursing experience (years) |  |  |  | 8.6 | 6.3 |
| Tenure in current unit (years) |  |  |  | 5.2 | 4.4 |
| Shift pattern | Day shift only | 187 | 26.0 |  |  |
|  | Rotating shifts (incl. night) | 533 | 74.0 |  |  |
| Clinical unit type (J = 36) | Medical | 12 | 33.3 |  |  |
|  | Surgical | 10 | 27.8 |  |  |
|  | ICU / Emergency | 8 | 22.2 |  |  |
|  | Other (e.g., oncology, paediatrics) | 6 | 16.7 |  |  |

**Table S4. Path Coefficients and Bootstrap Results for the Reverse Mediation Model (Burnout → POS → Breakroom Culture)**

| **Path / Effect** | **Standardized Estimate (β)** | **SE** | **95% BCa CI** | ***P*-value** |
| --- | --- | --- | --- | --- |
| Direct paths |  |  |  |  |
| Burnout → POS | 0.32 | 0.06 | [0.21, 0.43] | < 0.001 |
| POS → Breakroom Culture | 0.08 | 0.05 | [–0.02, 0.18] | 0.124 |
| Burnout → Breakroom Culture (direct) | –0.05 | 0.04 | [–0.13, 0.03] | 0.215 |
| Indirect effect |  |  |  |  |
| Burnout → POS → Breakroom Culture | 0.03 | 0.02 | [–0.01, 0.07] | 0.142 |

Note: BCa CI = bias-corrected and accelerated bootstrap confidence interval (5,000 resamples). Burnout was modelled as a latent variable comprising low psychological well-being and disengagement. All paths were estimated at the within level; between-level paths were not estimated due to insufficient between-unit variance for breakroom culture when specified as an outcome. Model fit: χ²(130) = 310.56, CFI = 0.955, TLI = 0.947, RMSEA = 0.050, SRMRwithin = 0.048, SRMRbetween = 0.082. Fit indices are substantially inferior to those of the hypothesized model (see Table S5).

**Table S5. Comparison of Alternative Models (Robustness Checks)**

| **Model Description** | **χ² (df)** | **CFI** | **TLI** | **RMSEA [90% CI]** | **SRMR (within / between)** | **Comparison with Hypothesized Model** |
| --- | --- | --- | --- | --- | --- | --- |
| 1. Hypothesized Model (Moderated Mediation) | 235.17 (128) | 0.970 | 0.962 | 0.039 [0.031–0.047] | 0.040 / 0.055 | Baseline |
| 2. Direct Effects Only (no mediation, no moderation) | 415.83 (134) | 0.938 | 0.930 | 0.057 [0.050–0.064] | 0.051 / 0.098 | ΔCFI = –0.032, ΔRMSEA = +0.018 |
| 3. Mediation-Only (no cross-level moderation) | 265.41 (130) | 0.965 | 0.958 | 0.043 [0.036–0.051] | 0.042 / 0.061 | ΔCFI = –0.005, ΔRMSEA = +0.004 |
| 4. Reverse Mediation (Burnout → POS → Breakroom) | 310.56 (128) | 0.955 | 0.947 | 0.050 [0.043–0.057] | 0.048 / 0.082 | ΔCFI = –0.015, ΔRMSEA = +0.011 |
| 5. Team-Level Work Engagement as Level-2 Predictor (TWE → BrC → POS → Burnout) | 278.34 (130) | 0.963 | 0.955 | 0.041 [0.034–0.048] | 0.043 / 0.068 | ΔCFI = –0.007, ΔRMSEA = +0.002 |

Note: The hypothesised model (Model 1) exhibits the best fit across all indices, supporting the specified causal structure and the inclusion of the cross-level interaction. Bold text indicates terminology updated from the original manuscript ("Team Climate" → "Team-Level Work Engagement").

**Mplus Syntax for the Hypothesised Cross‑Level Moderated Mediation Model**

TITLE: 2-1-1 Moderated Mediation Model

Breakroom Culture (L1) -> POS (L1 & L2) -> Burnout (L1)

Team-Level Work Engagement (L2) moderates

BrC -> POS at between level

DATA: FILE = "nurse_data.dat";

VARIABLE: NAMES = unitID BrC POS LowWB Dis TWE age gender edu

exp tenure shift;

USEV = BrC POS LowWB Dis TWE;

CLUSTER = unitID;

BETWEEN = TWE; ! Level-2 moderator (team-level

! work engagement)

WITHIN = ; ! All other variables treated as L1

ANALYSIS: TYPE = TWOLEVEL RANDOM;

ESTIMATOR = MLR; ! Robust maximum likelihood

BOOTSTRAP = 5000; ! Bootstrap resamples for CIs

MODEL:

! ---------- WITHIN LEVEL (Level 1) ----------

%WITHIN%

POS ON BrC (bw); ! Within effect of BrC on POS

LowWB ON POS (b1); ! Within effect of POS on low

! psychological well-being

LowWB ON BrC (c1_direct); ! Direct effect (control)

Dis ON POS (b2); ! Within effect of POS on

! disengagement

Dis ON BrC (c2_direct); ! Direct effect (control)

! ---------- BETWEEN LEVEL (Level 2) ----------

%BETWEEN%

! Latent decomposition (observed variables as single indicators)

BrC_BY BY BrC@1; ! Between component of BrC

POS_BY BY POS@1; ! Between component of POS

LowWB_BY BY LowWB@1; ! Between component of low WB

Dis_BY BY Dis@1; ! Between component of disengagement

! Structural paths at between level

POS_BY ON BrC_BY (bb); ! Between effect BrC → POS

POS_BY ON TWE; ! Main effect of team-level work

! engagement

! Cross-level interaction (BrC_BY × TWE)

INT_BrC_TWE | BrC_BY XWITH TWE;

POS_BY ON INT_BrC_TWE (gamma); ! Interaction effect

! Outcome paths at between level

LowWB_BY ON POS_BY (bbetween1);

Dis_BY ON POS_BY (bbetween2);

! Optional direct between effects (control)

LowWB_BY ON BrC_BY;

Dis_BY ON BrC_BY;

MODEL CONSTRAINT:

! Within indirect effects

NEW(ind_w_lowwb ind_w_dis);

ind_w_lowwb = bw * b1;

ind_w_dis = bw * b2;

! Between indirect effects (unconditional)

NEW(ind_b_lowwb ind_b_dis);

ind_b_lowwb = bb * bbetween1;

ind_b_dis = bb * bbetween2;

! Conditional indirect effects at low (-1SD), mean, high (+1SD) TWE

! Replace 4.05 and 0.71 with actual sample mean and SD of TWE

NEW(IND_LOW_LOWWB IND_MEAN_LOWWB IND_HIGH_LOWWB

IND_LOW_DIS IND_MEAN_DIS IND_HIGH_DIS);

IND_LOW_LOWWB = (bb + gamma*(-0.71)) * bbetween1;

IND_MEAN_LOWWB = (bb + gamma*0) * bbetween1;

IND_HIGH_LOWWB = (bb + gamma*0.71) * bbetween1;

IND_LOW_DIS = (bb + gamma*(-0.71)) * bbetween2;

IND_MEAN_DIS = (bb + gamma*0) * bbetween2;

IND_HIGH_DIS = (bb + gamma*0.71) * bbetween2;

! Index of moderated mediation

NEW(mod_index_lowwb mod_index_dis);

mod_index_lowwb = gamma * bbetween1;

mod_index_dis = gamma * bbetween2;

OUTPUT: TECH1 TECH8 STDYX CINTERVAL(BCBOOTSTRAP);

Reference: Preacher KJ, Zyphur MJ, Zhang Z. A general multilevel SEM

framework for assessing multilevel mediation. Psychological Methods.

2010;15:209–33.

The hypothesised model (Model 1) exhibits the best fit, supporting the

specified causal structure and the inclusion of the cross-level interaction.

**Supplementary Material 3 – Extended Theoretical Propositions and Supplementary Analyses**

**Description:** This supplementary material contains: (1) supplementary validation comparing indirect effect patterns using WHO-5 versus MBI-GS emotional exhaustion subscale; (2) shift pattern supplementary moderation analyses; (3) sensitivity analysis excluding covariates; and (4) de-identified correlation and covariance matrix availability statement.

**Section A: Supplementary Validation — Comparison of Indirect Effect Patterns Using WHO-5 vs. MBI-GS Emotional Exhaustion Subscale**

**A.1 Purpose**

This supplementary analysis was conducted in direct response to a reviewer's concern regarding whether the indirect effect pattern reported in the main study is robust to the choice of well-being versus burnout-specific instruments. Although the WHO-5 correlates strongly with the MBI-GS emotional exhaustion subscale (r = –0.73, P < 0.001), a high bivariate correlation does not guarantee equivalent mediation results. This analysis empirically tests whether the same "breakroom culture → POS → outcome" indirect effect pattern emerges when the outcome is operationalised using reverse-scored WHO-5 versus the MBI-GS emotional exhaustion subscale.

**A.2 Sample**

The analysis uses data from the 120-nurse validation subsample described in the main text (Methods 2.3). This subsample was drawn from the same three tertiary hospitals as the main study but was independent of the primary analysis sample (N = 720). Both the WHO-5 and the MBI-GS emotional exhaustion subscale (5 items) were administered to all 120 nurses. Demographic characteristics of this subsample were comparable to those of the main sample (female: 91.7%; mean age: 33.1 years, SD = 7.4; mean nursing experience: 9.0 years, SD = 6.5).

**A.3 Instrument Copyright Statement**

The MBI-GS is a copyrighted instrument (Mind Garden, Inc.). Permission for its use in the validation subsample was obtained under the ethical approval governing the original data collection (Jinzhou Medical University, Approval No: JZMULL2025328). The MBI-GS items are not reproduced in this supplementary material. Researchers seeking to use the MBI-GS should contact Mind Garden, Inc. (www.mindgarden.com) for licensing information. We report only de-identified statistical results derived from the collected data. The MBI-GS is correctly cited in the manuscript as Maslach, C., Jackson, S. E., & Leiter, M. P. (1996). Maslach Burnout Inventory manual (3rd ed.). Consulting Psychologists Press.

**A.4 Analytic Strategy**

We estimated the identical "breakroom culture → POS → outcome" mediation model twice on the same 120 nurses:

**Model 1:** Outcome = reverse-scored WHO-5 (low psychological well-being)

**Model 2:** Outcome = MBI-GS emotional exhaustion subscale

Both models were estimated using Mplus 8.6 with robust maximum likelihood (MLR) estimation and 5,000 bias-corrected bootstrap resamples. Age, gender, education, nursing experience, unit tenure, and shift pattern were included as covariates in both models. The indirect effects (ab) from the two models were formally compared using the MODEL CONSTRAINT command to compute the difference (Δab = ab_who5 − ab_mbi) and its 95% bias-corrected bootstrap confidence interval.

**A.5 Results**

**Descriptive Statistics and Correlation (N = 120)**

| **Variable** | **M** | **SD** | **1** | **2** | **3** | **4** |
| --- | --- | --- | --- | --- | --- | --- |
| 1. Breakroom Culture | 3.81 | 0.78 | — |  |  |  |
| 2. POS | 3.74 | 0.83 | 0.43*** | — |  |  |
| 3. WHO-5 (Reverse-scored) | 3.02 | 0.81 | –0.37*** | –0.50*** | — |  |
| 4. MBI-GS Emotional Exhaustion | 3.15 | 0.85 | –0.34*** | –0.47*** | 0.73*** | — |

Note: ****P* < 0.001.

**Parallel Mediation Model Results**

| **Parameter** | **WHO-5 Model (Reverse-scored)** | | | **MBI-GS Exhaustion Model** | | |
| --- | --- | --- | --- | --- | --- | --- |
|  | ***β*** | ***SE*** | ***95% BCa CI*** | ***β*** | ***SE*** | ***95% BCa CI*** |
| a path (BrC → POS) | 0.41*** | 0.08 | [0.25, 0.57] | 0.41*** | 0.08 | [0.25, 0.57] |
| b path (POS → Outcome) | –0.48*** | 0.08 | [–0.63, –0.33] | –0.46*** | 0.08 | [–0.61, –0.31] |
| c' path (BrC → Outcome, direct) | –0.06 | 0.07 | [–0.19, 0.07] | –0.04 | 0.07 | [–0.17, 0.10] |
| **Indirect effect (ab)** | **–0.20*** | **0.04** | **[–0.29, –0.12]** | **–0.18*** | **0.04** | **[–0.27, –0.10]** |
| Total effect (c) | –0.26*** | 0.08 | [–0.41, –0.11] | –0.22** | 0.08 | [–0.37, –0.07] |

Note: **P* < 0.05, ***P* < 0.01, ****P* < 0.001. BCa CI = bias-corrected and accelerated bootstrap confidence interval (5,000 resamples). All models controlled for age, gender, education, nursing experience, unit tenure, and shift pattern. BrC = Breakroom Culture; POS = Perceived Organisational Support. The a path is identical across models because both models use the same predictor and mediator variables estimated on the same sample

**Model Fit**

| **Model** | **χ²** | **df** | **CFI** | **RMSEA** | **SRMR** |
| --- | --- | --- | --- | --- | --- |
| WHO-5 Model | 18.42 | 16 | 0.98 | 0.04 | 0.04 |
| MBI-GS Model | 20.15 | 16 | 0.97 | 0.05 | 0.04 |

**Formal Test of Indirect Effect Difference**

| **Comparison** | **Δab** | **SE** | **95% BCa CI** | **Conclusion** |
| --- | --- | --- | --- | --- |
| WHO-5 ab − MBI-GS ab | –0.02 | 0.03 | [–0.07, 0.04] | Not significant |

The difference between the two indirect effects was not statistically significant (Δab = –0.02, 95% BCa CI [–0.07, 0.04]). The confidence interval includes zero, indicating that the magnitude of the indirect effect does not differ materially depending on whether the outcome is operationalised as low psychological well-being (WHO-5) or occupational exhaustion (MBI-GS).

**A.6 Interpretation**

Three key findings emerge from this supplementary validation:

**Directional consistency:** Both models yielded significant negative indirect effects via POS. The sign, significance, and overall pattern of mediation are identical across the two operationalisations.

**Magnitude similarity:** The indirect effect estimates are closely comparable (–0.20 vs. –0.18), and their 95% confidence intervals substantially overlap ([–0.29, –0.12] vs. [–0.27, –0.10]). The formal test confirms that the difference is not statistically significant.

**Pattern replication:** In both models, the direct effect of breakroom culture on the outcome was non-significant, and the indirect effect via POS was significant—mirroring the pattern reported in the full sample (N = 720).

These results provide empirical evidence that the informal-organisational support pathway is not an artefact of using a well-being instrument rather than a burnout-specific instrument. The same theoretical pathway emerges whether the outcome is operationalised using the WHO-5 or the MBI-GS—the most widely recognised international standard for burnout assessment.

**A.7 Mplus Syntax for Parallel Mediation Models and Indirect Effect Comparison**

TITLE: Supplementary Validation — Parallel Mediation Models

WHO-5 (Reverse-scored) vs. MBI-GS Emotional Exhaustion

N = 120 validation subsample

NOTE: 'WHO5_r' = reverse-scored WHO-5 Well-Being Index

(higher scores = lower well-being/higher exhaustion-related distress)

NOTE: 'MBI_EE' = MBI-GS Emotional Exhaustion subscale

(MBI-GS is a copyrighted instrument; items are not reproduced)

DATA: FILE = "validation_subsample.dat";

VARIABLE: NAMES = id BrC POS WHO5_r MBI_EE age gender edu exp tenure shift;

USEV = BrC POS WHO5_r MBI_EE age gender edu exp tenure shift;

ANALYSIS: BOOTSTRAP = 5000;

ESTIMATOR = MLR;

MODEL:

! Common a path (same predictor, same mediator)

POS ON BrC (a);

! Model 1: WHO-5 (reverse-scored) as outcome

WHO5_r ON POS (b1);

WHO5_r ON BrC (c1);

! Model 2: MBI-GS Emotional Exhaustion as outcome

MBI_EE ON POS (b2);

MBI_EE ON BrC (c2);

! Covariates

POS ON age gender edu exp tenure shift;

WHO5_r ON age gender edu exp tenure shift;

MBI_EE ON age gender edu exp tenure shift;

MODEL CONSTRAINT:

! Indirect effects

NEW(ab_who5 ab_mbi diff_ab);

ab_who5 = a * b1;

ab_mbi = a * b2;

diff_ab = ab_who5 - ab_mbi;

OUTPUT: STDYX CINTERVAL(BCBOOTSTRAP);

**Note:** This syntax was adapted from the main study's Mplus code (see Supplementary Material 2). Variable names: BrC = Breakroom Culture; POS = Perceived Organisational Support; WHO-5_r = Reverse-scored WHO-5; MBI_EE = MBI-GS Emotional Exhaustion subscale. The actual sample mean and standard deviation values should be inserted in MODEL CONSTRAINT for conditional effect calculations if needed.

**Section B: Shift Pattern Supplementary Analyses**

Given that 74% of the main sample worked rotating shifts (including nights), we conducted supplementary analyses to examine whether shift pattern moderated any of the hypothesised paths. Shift pattern (day-only = 0, rotating = 1) was entered as a moderator of the breakroom culture → POS and POS → burnout (low psychological well-being and disengagement) paths.

**Results:**

| **Interaction Term** | ***β*** | ***SE*** | ***P*** |
| --- | --- | --- | --- |
| Breakroom culture × shift pattern → POS | –0.03 | 0.04 | 0.48 |
| POS × shift pattern → Low psychological well-being | 0.02 | 0.04 | 0.62 |
| POS × shift pattern → Disengagement | 0.01 | 0.04 | 0.79 |

No significant interaction effects were detected. The pattern of conditional indirect effects remained stable when the model was estimated separately for day-only and rotating shift subsamples. These findings suggest that the protective associations of breakroom culture and POS operate similarly across shift types.

**Section C: Sensitivity Analysis — Exclusion of Covariates**

The full moderated mediation model was re-estimated after omitting all covariates (age, gender, education, nursing experience, unit tenure, shift pattern). All path coefficients, indirect effects, and the cross-level interaction term remained stable in direction, magnitude, and statistical significance. These results confirm that the observed associations are not driven by the specific set of covariates included in the main model.

**Section D: De-Identified Correlation and Covariance Matrices**

Due to institutional ethics restrictions, the raw individual-level dataset cannot be publicly deposited. The within-level and between-level correlation matrices and covariance matrices sufficient to reproduce the main structural model results are available from the corresponding author upon reasonable request and after approval from the participating hospitals' ethics committees.

**Supplementary Material 4 – Prototype Breakroom Audit Checklist**

Description: A brief checklist for nurse managers to annually assess the

physical and psychosocial conditions of staff breakrooms. This tool is

intended as a practical, adaptable resource derived from the study's

findings and the broader recovery environment literature (Sonnentag &

Fritz, 2015; Connors et al., 2024). It has not been formally validated

and should be adapted to fit local contexts.

**BREAKROOM AUDIT CHECKLIST**

Unit: ____________________ Date: ____ / ____ / ______

Assessed by: ____________________

Instructions: For each item, indicate whether the criterion is Met, Partially Met, or Not Met. Add comments where relevant.

A. PHYSICAL ENVIRONMENT

**Item Met Partial Not Met Comments**

(1) Adequate seating is available for the number of staff using the breakroom during peak times.
(2) The breakroom has access to natural light or adequate artificial lighting.
(3) A quiet zone or area is designated for psychological detachment (away from phones, pagers, or clinical conversation).
(4) Comfortable seating (e.g., chairs with back support, soft seating options) is available.
(5) The breakroom is clean, well-maintained, and free from clinical equipment or supplies.
(6) Basic amenities (e.g., tea/coffee facilities, refrigerator, microwave, drinking water) are available and functional.

B. PSYCHOSOCIAL ENVIRONMENT

Item Met Partial Not Met Comments

(7) Staff generally use the breakroom for genuine rest and recovery during break periods (rather than for administrative tasks or meetings).
(8) Informal peer support and collegial conversation are visibly present during break times.
(9) Negative interpersonal behaviours (e.g., incivility, gossip, conflict) are infrequent in the breakroom.
(10) Staff feel psychologically safe to express work-related difficulties or emotions in the breakroom.
(11) The breakroom atmosphere is perceived as warm and supportive (based on informal staff feedback).

C. ORGANISATIONAL SUPPORT

Item Met Partial Not Met Comments

(12) Written policies exist that protect break time from non-clinical, non-educational chores.
(13) Managers or supervisors are occasionally present in the breakroom in an informal, supportive capacity.
(14) Peer support in informal settings is recognised or rewarded (e.g., in team meetings, recognition boards).

D. OVERALL ASSESSMENT

Overall, the breakroom serves as a restorative

space contributing to staff well-being.

Summary Score: Met: ____ /15 Partial: ____ /15 Not Met: ____ /15

Action Items Identified:

Review Date for Follow-Up: ____ / ____ / ______

Note: This checklist is a prototype developed for managerial application

based on the study's conceptual framework and the broader recovery

environment literature. It has not undergone formal psychometric validation.

Users are encouraged to adapt it to their specific clinical contexts.

**Supplementary Material 5 – Cross-Cultural Hypotheses for Future Research**

(Formerly Presented in Discussion 4.1 of Original Draft)

The following cross-cultural research propositions were formally presented in the Discussion section of the original manuscript. In the revised manuscript, these have been condensed for brevity and moved here to supplementary materials. They are preserved in full to guide future comparative investigations.

Background: The informal-organisational support pathway (breakroom culture→ POS → reduced burnout) is theorised to be particularly salient in contexts characterised by collectivism and high power distance, such as China, where informal relational networks (guanxi) serve as a fundamental social currency. Cross-cultural comparative studies are needed to empirically verify whether this pathway is indeed stronger in such contexts than in more individualistic or low-power-distance settings.

Hypothesis 1 (Cultural Moderation of the Indirect Effect):

The indirect effect of breakroom culture on burnout (low psychological well-being and disengagement) via POS will be stronger in high-power-distance cultures than in low-power-distance cultures.

Rationale: In high-power-distance contexts, frontline employees perceive a greater distance from formal organisational authority. Supportive peer interactions in informal spaces may therefore function as a more critical, accessible proxy for organisational care, partially compensating for perceived deficiencies in top-down support. In low-power-distance contexts, employees may have more direct access to formal support channels, reducing the relative importance of the informal pathway.

Hypothesis 2 (Cultural Moderation of the First-Stage Moderation):

The moderating effect of team-level work engagement on the breakroom culture–POS relationship will be more pronounced in collectivistic than in individualistic contexts.

Rationale: In collectivistic cultures, consistency between informal peer support (breakroom) and formal team environment (team work engagement) carries greater normative weight. The synergistic reinforcement of congruent support signals—both informal and formal—is more likely to be interpreted as genuine organisational care. In individualistic contexts, employees may weigh these signals more independently, reducing the synergy effect.

Suggested Design:

Multi-nation data collection spanning at least two countries that differ systematically on power distance and individualism–collectivism dimensions (e.g., China vs. Netherlands vs. United States).

Measurement invariance testing across cultural groups prior to hypothesis testing.

Multilevel moderated mediation with cultural values (country-level or individually measured) as a cross-cultural moderator of the indirect pathway.

Testing these propositions would significantly extend the theoretical generalisability of the informal-organisational support pathway and determine the boundary conditions under which breakroom culture enhancements are most effective.
